# Management of Patients With COVID-19 During the Pandemic: A Retrospective Cohort Study From a Tertiary Care Centre in Mumbai, India

**DOI:** 10.7759/cureus.83711

**Published:** 2025-05-08

**Authors:** Neeraj Tulara, Swapnil Mehta, Vimal Pahuja, Shalini Suralkar, Arpita Dwivedy, Maheema Bhaskar, Sajit Babu, Archana Chitnis, Suvin Shetty, Maninder S Setia

**Affiliations:** 1 Internal Medicine, Dr. LH Hiranandani Hospital, Mumbai, IND; 2 Pulmonology, Dr. LH Hiranandani Hospital, Mumbai, IND; 3 Intensive Care, Dr. LH Hiranandani Hospital, Mumbai, IND; 4 Microbiology, Dr. LH Hiranandani Hospital, Mumbai, IND; 5 Pathology, Dr. LH Hiranandani Hospital, Mumbai, IND; 6 Epidemiology, Dr. LH Hiranandani Hospital, Mumbai, IND

**Keywords:** clinical experience, cohort, covid-19, doxycycline, enoxaparin, mortality, remdesivir, tocilizumab

## Abstract

Introduction: We conducted the present analysis to understand the clinical outcomes in patients with COVID-19 infection and the factors associated with mortality over the one-and-a-half years of the pandemic in Mumbai, India.

Methods: This is a retrospective cohort study of 3561 COVID-19 patients admitted to a private tertiary care hospital in Mumbai, India. The main outcome variable for analysis was death (yes/no). We compared the characteristics of COVID-19 patients who had died during their stay in the hospital with those who had not died. We used survival analysis to identify the factors associated with mortality.

Results: The mortality rate was 0.71 per 100 person-days (95% CI: 0.61-0.81 per 100 person days (PD)); it was higher (2.03 (95% CI: 1.47, 2.82) per 100 PD) in those aged >= 80 years compared with 18-39 years (p<0.001). The hazard rate (HR) was significantly higher in those with chronic liver disease (HR: 5.12, 95% CI: 1.78, 14.71; p=0.002) and lower in those who were treated with injection enoxaparin (HR: 0.46, 95% CI: 0.31, 0.69; p<0.001). In patients without breathing difficulties, the hazard was significantly lower in those treated with doxycycline (HR: 0.41, 95% CI: 0.17, 0.99; p=0.05). In patients who presented with breathing difficulties, hazard was significantly lower in those who were treated with remdesivir (HR: 0.52, 95% CI: 0.31, 0.90; p=0.019). In patients who required ventilatory support (invasive and non-invasive), the hazard was significantly lower with injected tocilizumab (HR: 0.56, 95% CI: 0.36, 0.89; p=0.013).

Conclusions: The mortality was highest during the initial days of the pandemic and in patients with co-morbidities. Remdesivir and the tocilizumab injection were useful in the reduction of mortality in the severe form of COVID-19 infection. Doxycycline was useful in milder and less severe forms of infection. However, enoxaparin injection was associated with lower mortality in most of these cases.

## Introduction

Some initial reports of pneumonia clusters were reported in late December 2019 in China, with the World Health Organization monitoring the situation closely [1]. In January 2020, the WHO acknowledged that there may be human-to-human transmission and released an online free course on the novel coronavirus [1]. The International Committee on Taxonomy of Viruses named the virus severe acute respiratory syndrome coronavirus 2 (SARS-CoV-2) and the disease, coronavirus disease (COVID-19) on 11 February 2020 [2]. In India, the first case of COVID-19 was reported in January 2020 from Kerala in an individual who had returned from China [3]. Finally, on March 11, 2020, the WHO declared COVID-19 to be a pandemic, and the Government of India declared a nationwide lockdown on March 24, 2020. The Government of India and the Indian Council for Medical Research (ICMR) released regular guidelines on diagnosis, quarantine procedures, and management of COVID-19, and epidemiological data from the country [4-6].

Given the nature of the pandemic, many management strategies were discussed early on in the pandemic globally. These included existing drugs such as antimalarials, chloroquine and hydroxychloroquine, ivermectin, and antibiotics (such as azithromycin and doxycycline) [7-12]. Though there were some controversies around the use of these medications, many countries included them in their guidelines for the management of COVID-19 infections [7,10]. The next set of medications was antivirals such as remdesivir (nucleoside analogs) or protease inhibitors (such as lopinavir or ritonavir) [7,13,14]. Steroids and biologics (tocilizumab) were also used for the management of COVID-19-related symptoms and complications [7,10]. Anticoagulants were the other group of medications recommended and used in the infection; this was mainly due to the coagulopathies associated with COVID-19 disease and mortality. Various studies have used heparin, warfarin, and enoxaparin for the management of COVID-19, particularly in patients with increased D-dimer and fibrinogen [15,16]. Besides these medications, immunoglobulins, interferons, and convalescent plasma were also used, and reports were published during the early months of the pandemic [10,17]. Other treatment modalities, such as zinc supplements, ayurvedic, and homeopathic medicines, were also used in the management of COVID-19 patients [18-21]. Oxygen therapy, ventilation, and supportive therapy were also useful in cases of severe COVID-19 infection [22,23].

Though there were reports and recommendations, many clinicians also treated the patients based on new clinical reports and their own experience treating patients [24,25]. Some of the published reports presented contradictory findings. In many cases, a combination of medications was used, and the sequence of treatment changed based on the clinical features and laboratory findings. During the active phase of the pandemic, a lot of case reports, studies, and trials were published. They helped in changing and improving the management of COVID-19 patients during the multiple waves of the pandemic in India. Since the active pandemic is pretty much behind us, an analysis of the experience will help us identify the therapies that worked and the ones that didn’t over time for the management of those infected with COVID-19.

The current analysis was carried out in Mumbai, India, to assess the clinical outcomes of patients infected with COVID-19 and to investigate the parameters linked to mortality during the pandemic's one and a half-year duration.

## Materials and methods

This is a retrospective cohort study (secondary data analysis) from 3561 COVID-19 patients admitted from March 2020 through December 2021.

Study site, participants, and variables

The study was conducted at Dr. L. H. Hiranandani Hospital, Powai, India. It is a tertiary care private hospital situated in suburban Mumbai and was one of the hospitals responsible for the management of COVID-19 patients from the beginning of the pandemic (March 2020) in India. All the patients were admitted to a special COVID-19 ward created in the hospital. Patients who were serious and required additional management were shifted to the intensive care unit of the hospital. All the patients were managed according to the existing clinical and national guidelines (ICMR guidelines).

We abstracted the following data from the clinical records for the present analysis: demographic information (age, gender); risk factors for COVID-19 (since we collected data from the beginning of the pandemic, history of risk factors such as travel, contact with known COVID-19, and health care worker was an important component of record); presenting complaints (such as fever, breathlessness, anosmia, etc.); co-morbidities (such as diabetes mellitus, hypertension, etc.); treatment given/medications received by the patients (hydroxychloroquine, remdesivir, prednisolone, tocilizumab, etc.); oxygen requirement and type of oxygen required); days of admission to the hospital, including the ICU; and outcome (discharge/death).

Statistical methods

We estimated the means and standard deviations (SDs) or medians and interquartile range for linear variables. We estimated the proportions for categorical variables. The main outcome variable for cross-tabulation was death (yes/no). We compared the characteristics in COVID-19 patients who had died during their stay in the hospital with those who had not died. The proportions across groups were compared using the chi-square test or Fisher’s exact test for low expected cell counts.

Following this, we used survival analysis models for further analysis. We estimated the mortality rate in these patients (overall and according to various characteristics). We plotted the Kaplan-Meier survival curves to estimate the survival. We used the log-rank test to assess the equality of the survivor function across various categories. We then used the Cox proportional hazard models to estimate the hazard ratio (HR) and its 95% confidence intervals. We built univariate and multivariate models. The fit of the models was assessed using the Akaike Information Criteria and Bayesian Information Criteria. After the model with the whole study population, we built individual models for those who presented with breathlessness vs. those who did not; those who required oxygen vs. those who did not require oxygen; and those who required ventilation (invasive/non-invasive) vs. those who did not require it. A p-value of <0.05 was considered statistically significant.

The Dr. L H Hiranandani Hospital's Ethics Committee approved the study with reference number DRLHH/EC/2023/15 on August 1, 2023. We used anonymized data from clinical records for the present secondary data analysis.

## Results

**Figure 1:**
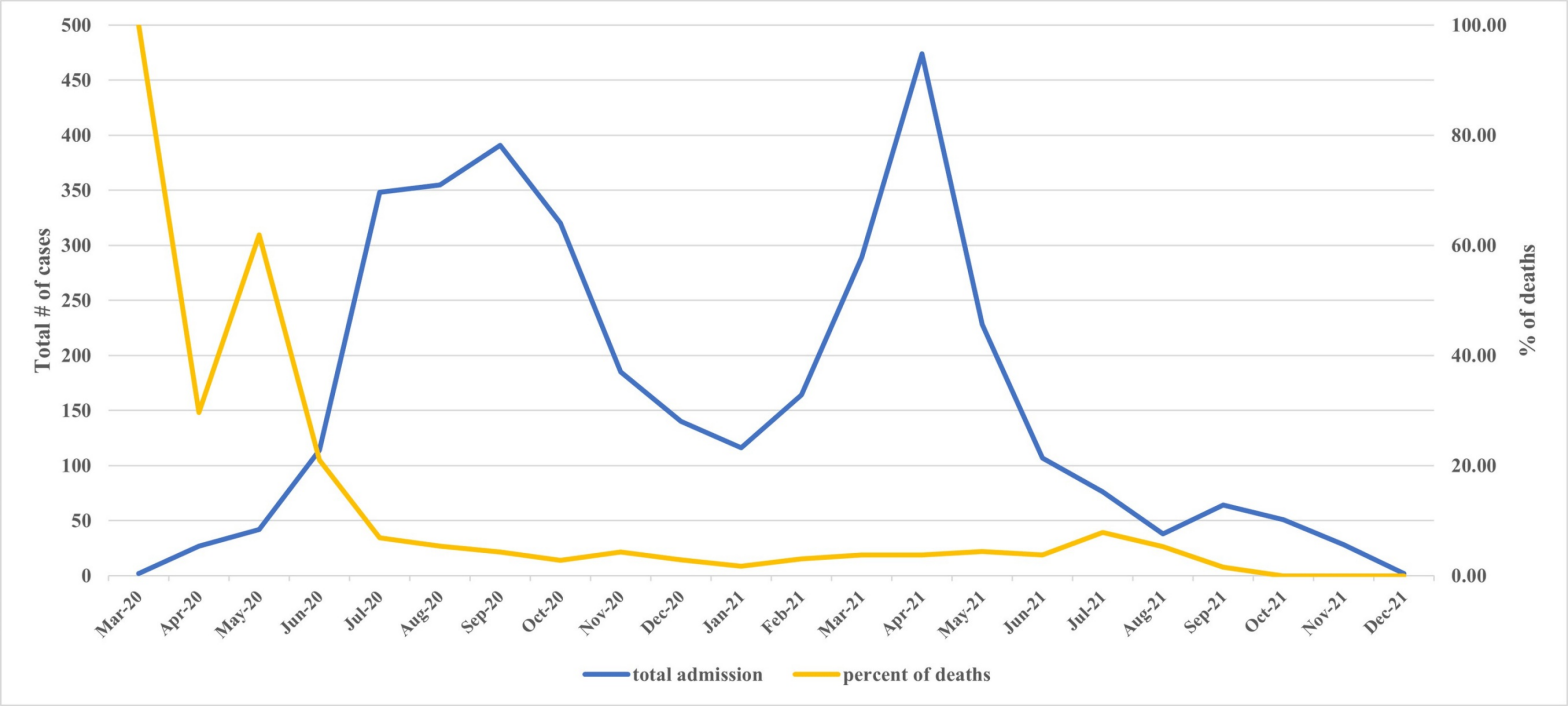
Total number of cases and percentage of deaths in 3561 patients with COVID-19 admitted to a tertiary care hospital in Mumbai, India

**Figure 2:**
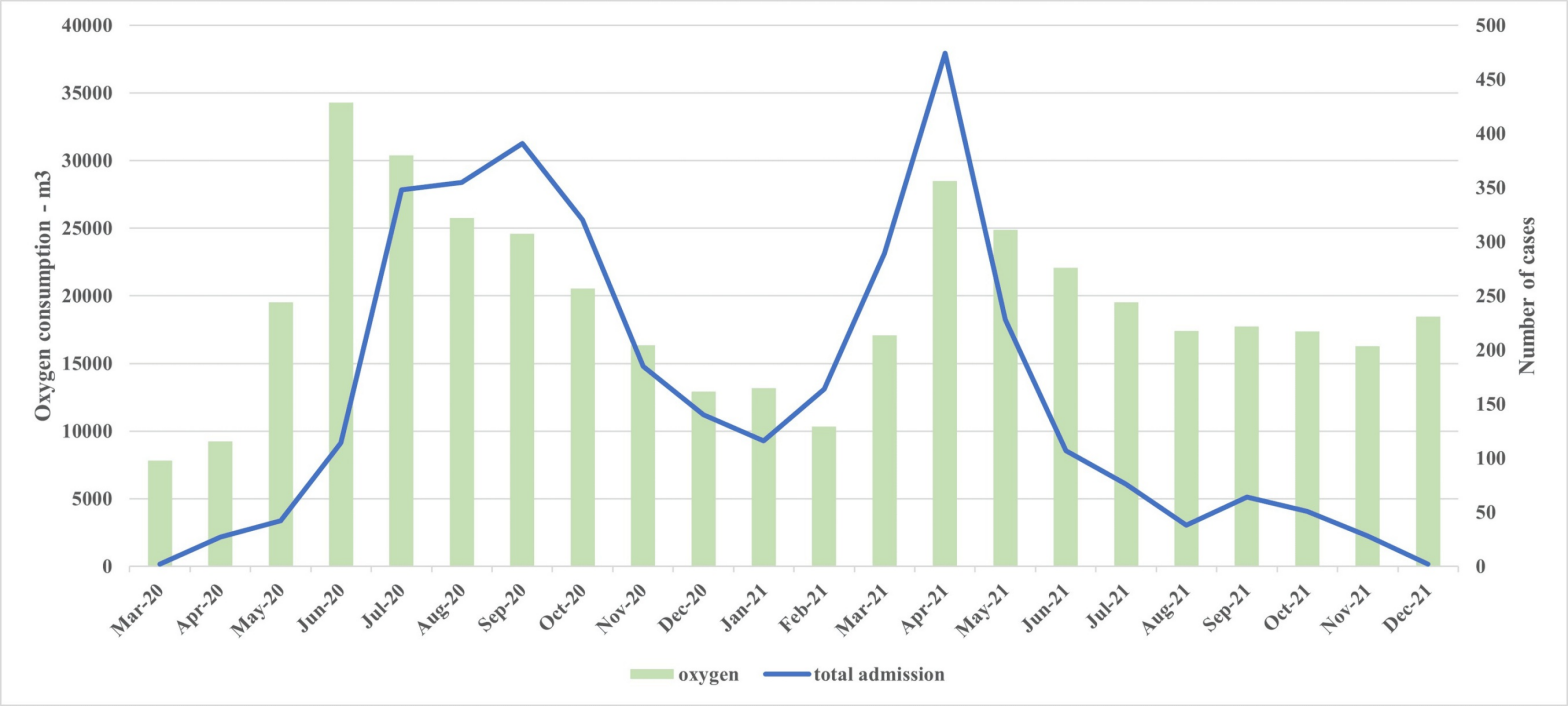
Total number of cases and oxygen requirement in 3561 patients with COVID-19 admitted to a tertiary care hospital in Mumbai, India

The total number of deaths over this 22-month period was 200; the proportion of deaths was 5.6% (n=200), and the mortality rate was 0.71 per 100 person-days (95% CI: 0.61-0.81 per 100 PD). The number of patients varied over time; it was maximum during the initial two waves (June 2020 through October 2020), followed by a sharp peak from March 2021 through June 2021 (Figure 1). These waves also corresponded with the waves of the pandemic in the city of Mumbai and India. The proportion of deaths was very high in the initial months of the pandemic. Indeed, only two patients were admitted in the month of March 2020, and both died. However, the proportion of deaths substantially decreased over the next couple of months and remained more or less similar till December 2021 (but for a slight increase in June and July 2021) (Figure 1). The oxygen requirement increased during these months. The total oxygen consumption in the hospital was 7810 cubic meters in March 2020. It increased to a maximum value of 34288 m³ in May 2020; the change in oxygen use nearly corresponded with the total number of admissions (Figure 2).

The mean (SD) age of patients was 56.1 (15.4) years. Most of the patients were in the age group of 50-69 years and were males (64.6% (n=2302)) (Table 1). About 3.2% (n=115) were health care providers, 4.6% (n=164) had a history of travel, and 13.2% (n=471) had a history of contact with a patient with COVID-19. The most common presenting features were fever (79.6% (n=2835)), cough (63.5% (n=2262)), breathing difficulties (29.3% (n=1043)), muscle pain (21.9% (n=780)), headache (18.2% (n=648)), and soreness in the body (16.3% (n=581)). Other symptoms, such as loss of smell (11.3% (n=401)), rash on the body (0.2% (n=6)), and redness of the eyes (0.5% (n=18)), were less commonly observed. The most common comorbidities in our patients were hypertension (40.2% (n=1432)) and diabetes mellitus (34.8% (n=1239)). The treatment given to the patients changed over time. Hydroxychloroquine (HCQ) was used in the early months of the pandemic; however, there was a gradual increase in the use of remdesivir and injection enoxaparin. The demographic factors associated with mortality were age (70 years and above) and male gender (Table 1). The proportion of deaths was significantly higher in those who presented with breathing difficulties compared with those who did not (12.2% (n=127) vs. 2.9% (n=73); p<0.001) (Table 1). The proportion of deaths was higher in patients with diabetes mellitus (7.8% (n=97) vs. 4.4% (n=103); p<0.001), hypertension (8.4% (n=120) vs. 3.8% (n=80); p<0.001), and chronic kidney disease (31.8% (n=27) vs. 5.0% (n=173); p<0.001) (Table 2). The proportion of deaths was lower in those who were treated with remdesivir (4.3% (n=102) vs. 8.4% (n=98); p<0.001) and enoxaparin injection (4.2% (n=129) vs. 13.5% (n=71); p<0.001) (Table 3). We also found that the proportion of deaths was higher in those who required oxygen compared with those who did not (18.4% (n=193) vs. 0.3% (n=7); p<0.001); particularly those who required non-invasive ventilation support (85.0% (n=113) vs. 2.5% (n=87); p<0.001) and invasive ventilation support (90.9% (n=20) vs. 5.1% (n=180); p<0.001) (Table 3). We have presented detailed proportions in Tables 1, 2, 3.

**Table 1:** Proportion of deaths according to demographic characteristics and presenting features in 3561 patients admitted with COVID-19 in a tertiary care hospital in Mumbai, India The data are presented as numbers (N) and percentages (%) (in parentheses). The total column shows column percentages, and the other two (death-yes and no) show row percentages. The test statistic represents the chi-square value. A p-value of <0.05 was considered statistically significant. * chi-square for trend.

Characteristics	Total	Death: Yes	Death: No	Test statistic	P-value
	N (%)	n (%)	n (%)		
All	3561 (100.0)	200 (5.6)	3361 (94.4)		
Demographic data					
Age groups					
18-29	147 (4.1)	3 (2.0)	144 (98.0)	121.41	< 0.001
30-39	434 (12.2)	7 (1.6)	427 (98.4)	93.59*	< 0.001*
40-49	651 (18.3)	16 (2.5)	635 (97.5)		
50-59	768 (21.6)	32 (4.2)	736 (95.8)		
60-69	787 (22.1)	44 (5.6)	743 (94.4)		
70-79	580 (16.3)	62 (10.7)	518 (89.3)		
>=80	194 (5.4)	36 (18.6)	158 (81.4)		
Gender					
Female	1259 (35.4)	51 (4.1)	1208 (95.9)	9.00	0.003
Male	2302 (64.6)	149 (6.5)	2153 (93.5)		
Travel					
Missing	6 (0.2)	0 (0)	6 (100)	3.65	0.163
No	3391 (95.2)	196 (5.8)	3195 (94.2)		
Yes	164 (4.6)	4 (2.4)	160 (97.6)		
Contact					
Missing	7 (0.2)	0 (0)	7 (100)	8.84	0.011
No	3083 (86.6)	187 (6.1)	2896 (93.9)		
Yes	471 (13.2)	13 (2.8)	458 (97.2)		
Health care worker					
Missing	13 (0.4)	0 (0)	13 (100)	4.18	0.144
No	3433 (96.4)	198 (5.8)	3235 (94.2)		
Yes	115 (3.2)	2 (1.7)	113 (98.3)		
Clinical presentation					
Fever					
No	726 (20.4)	43 (5.9)	683 (94.1)	0.16	0.755
Yes	2835 (79.6)	157 (5.5)	2678 (94.5)		
Breathing difficulties					
No	2518 (70.7)	73 (2.9)	2445 (97.1)	119.75	< 0.001
Yes	1043 (29.3)	127 (12.2)	916 (87.8)		
Cough					
No	1299 (36.5)	75 (5.8)	1224 (94.2)	0.10	0.816
Yes	2262 (63.5)	125 (5.5)	2137 (94.5)		
Chills					
No	3397 (95.4)	197 (5.8)	3200 (94.2)	4.65	0.024
Yes	164 (4.6)	3 (1.8)	161 (98.2)		
Muscle pain					
No	2781 (78.1)	187 (6.7)	2594 (93.3)	29.39	< 0.001
Yes	780 (21.9)	13 (1.7)	767 (98.3)		
Soreness in the body					
No	2980 (83.7)	187 (6.3)	2793 (93.7)	14.95	< 0.001
Yes	581 (16.3)	13 (2.2)	568 (97.8)		
Headache					
No	2913 (81.8)	181 (6.2)	2732 (93.8)	10.77	0.001
Yes	648 (18.2)	19 (2.9)	629 (97.1)		
Diarrhea					
No	3259 (91.5)	187 (5.7)	3072 (94.3)	1.07	0.366
Yes	302 (8.5)	13 (4.3)	289 (95.7)		
Vomiting					
No	3406 (95.6)	191 (5.6)	3215 (94.4)	0.01	0.858
Yes	155 (4.4)	9 (5.8)	146 (94.2)		
Loss of smell					
No	3160 (88.7)	195 (6.2)	2965 (93.8)	16.28	< 0.001
Yes	401 (11.3)	5 (1.2)	396 (98.8)		
Rash on the body					
No	3555 (99.8)	200 (5.6)	3355 (94.4)	0.36	>0.99
Yes	6 (0.2)	0 (0)	6 (0.2)		
Redness of the eyes					
No	3543 (99.5)	199 (5.6)	3344 (94.4)	0.00	>0.99
Yes	18 (0.5)	1 (5.6)	17 (94.4)		

**Table 2:** Proportion of deaths according to co-morbidities in 3561 patients admitted with COVID-19 in a tertiary care hospital in Mumbai, India The data are presented as numbers (N) and percentages (%) (in parentheses). The total column shows column percentages, and the other two (death-yes and no) show row percentages. The test statistic represents the chi-square value. A p-value of <0.05 was considered statistically significant. COPD: chronic obstructive pulmonary disease

Co-morbidities	Total	Death: Yes	Death: No	Test statistic	P-value
	N (%)	n (%)	n (%)		
All	3561 (100.0)	200 (5.6)	3361 (94.4)		
Asthma					
No	3465 (97.3)	194 (5.6)	3271 (94.4)	0.07	0.657
Yes	96 (2.7)	6 (6.2)	90 (93.8)		
COPD					
No	3509 (98.5)	196 (5.6)	3313 (94.4)	0.43	0.535
Yes	52 (1.5)	4 (7.7)	48 (92.3)		
Diabetes mellitus					
No	2322 (65.2)	103 (4.4)	2219 (95.6)	17.55	< 0.001
Yes	1239 (34.8)	97 (7.8)	1142 (92.2)		
Hypertension					
No	2129 (59.8)	80 (3.8)	2049 (96.2)	34.51	< 0.001
Yes	1432 (40.2)	120 (8.4)	1312 (91.6)		
Chronic kidney disease					
No	3476 (97.6)	173 (5.0)	3303 (95)	112.32	< 0.001
Yes	85 (2.4)	27 (31.8)	58 (68.2)		
Heart disease					
No	3306 (92.8)	171 (5.2)	3135 (94.8)	17.17	< 0.001
Yes	255 (7.2)	29 (11.4)	226 (88.6)		
Chronic liver disease					
No	3548 (99.6)	196 (5.5)	3352 (94.5)	15.57	0.005
Yes	13 (0.4)	4 (30.8)	9 (69.2)		
Immunocompromised state					
No	3547 (99.6)	200 (5.6)	3347 (94.4)	0.84	>0.99
Yes	14 (0.4)	0 (0)	14 (100.0)		
Body mass index (kg/m^2^)					
<18.5 Underweight	19 (0.5)	2 (10.5)	17 (89.5)	46.66	<0.001
18.5-22.9 Normal	263 (7.4)	6 (2.3)	257 (97.7)		
23-25 Overweight	495 (13.9)	12 (2.4)	483 (97.6)		
>25 Obese	878 (24.7)	28 (3.2)	850 (96.8)		
Missing	1906 (53.5)	152 (8.0)	1754 (92.0)		

**Table 3:** Proportion of deaths according to treatment given and oxygen requirement in 3561 patients admitted with COVID-19 in a tertiary care hospital in Mumbai, India The data are presented as numbers (N) and percentages (%) (in parentheses). The total column shows column percentages, and the other two (death-yes and no) show row percentages. The test statistic represents the chi-square value. A p-value of <0.05 was considered statistically significant. NRBM: non-rebreather mask; VS NIV: noninvasive ventilation; VS invasive: invasive ventilation

Treatment given	Total	Death: Yes	Death: No	Test statistic	P-value
	N (%)	n (%)	n (%)		
All	3561 (100.0)	200 (5.6)	3361 (94.4)		
Ivermectin					
No	1163 (32.7)	90 (7.7)	1073 (92.3)	14.67	< 0.001
Yes	2398 (67.3)	110 (4.6)	2288 (95.4)		
Doxycycline					
No	1106 (31.1)	87 (7.9)	1019 (92.1)	15.32	< 0.001
Yes	2455 (68.9)	113 (4.6)	2342 (95.4)		
Remdesivir					
No	1163 (32.7)	98 (8.4)	1065 (91.6)	25.73	< 0.001
Yes	2398 (67.3)	102 (4.3)	2296 (95.7)		
Lopinavir/Ritonavir					
No	3354 (94.2)	148 (4.4)	3206 (95.6)	157.72	< 0.001
Yes	207 (5.8)	52 (25.1)	155 (74.9)		
Hydoxychloroquine					
No	2903 (81.5)	139 (4.8)	2764 (95.2)	20.33	<0.001
Yes	658 (18.5)	61 (9.3)	597 (90.7)		
Methylprednisolone					
No	1948 (54.7)	72 (3.7)	1876 (96.3)	29.92	< 0.001
Yes	1613 (45.3)	128 (7.9)	1485 (92.1)		
Injection tocilizumab					
No	3396 (95.4)	136 (4.0)	3260 (96)	359.14	< 0.001
Yes	165 (4.6)	64 (38.8)	101 (61.2)		
Injection enoxaparin					
No	525 (14.7)	71 (13.5)	454 (86.5)	72.63	< 0.001
Yes	3036 (85.3)	129 (4.2)	29.07 (95.8)		
Plasma therapy					
No	3510 (98.6)	190 (5.4)	3320 (94.6)	19.11	<0.001
Yes	51 (1.4)	10 (19.6)	41 (80.4)		
Oxygen requirement					
Oxygen required					
No	2510 (70.5)	7 (0.3)	2503 (99.7)	457.05	< 0.001
Yes	1051 (29.5)	193 (18.4)	858 (81.6)		
Nasal					
No	2748 (77.2)	131 (4.8)	2617 (95.2)	16.38	< 0.001
Yes	813 (22.8)	69 (8.5)	744 (91.5)		
NRBM					
No	3227 (90.6)	95 (2.9)	3132 (97.1)	463.56	< 0.001
Yes	334 (9.4)	105 (31.4)	229 (68.6)		
VS NIV					
No	3428 (96.3)	87 (2.5)	3341 (97.5)	1625.37	< 0.001
Yes	133 (3.7)	113 (85.0)	20 (15.0)		
VS invasive					
No	3539 (99.4)	180 (5.1)	3359 (94.9)	303.77	< 0.001
Yes	22 (0.6)	20 (90.9)	2 (9.1)		

Survival analysis and Cox proportional hazard models

**Figure 3:**
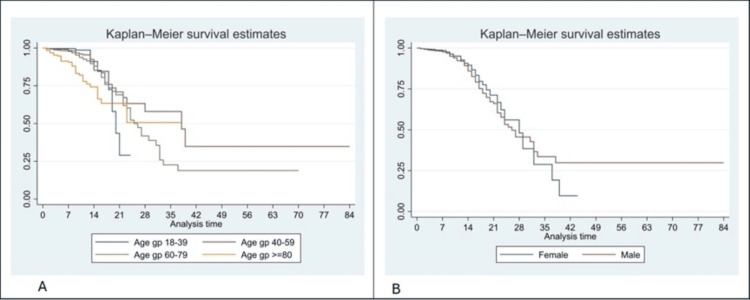
Kaplan-Meier survival curves in 3561 patients admitted with COVID-19 in a tertiary care hospital according to age groups (A) and gender (B) in Mumbai, India

**Figure 4:**
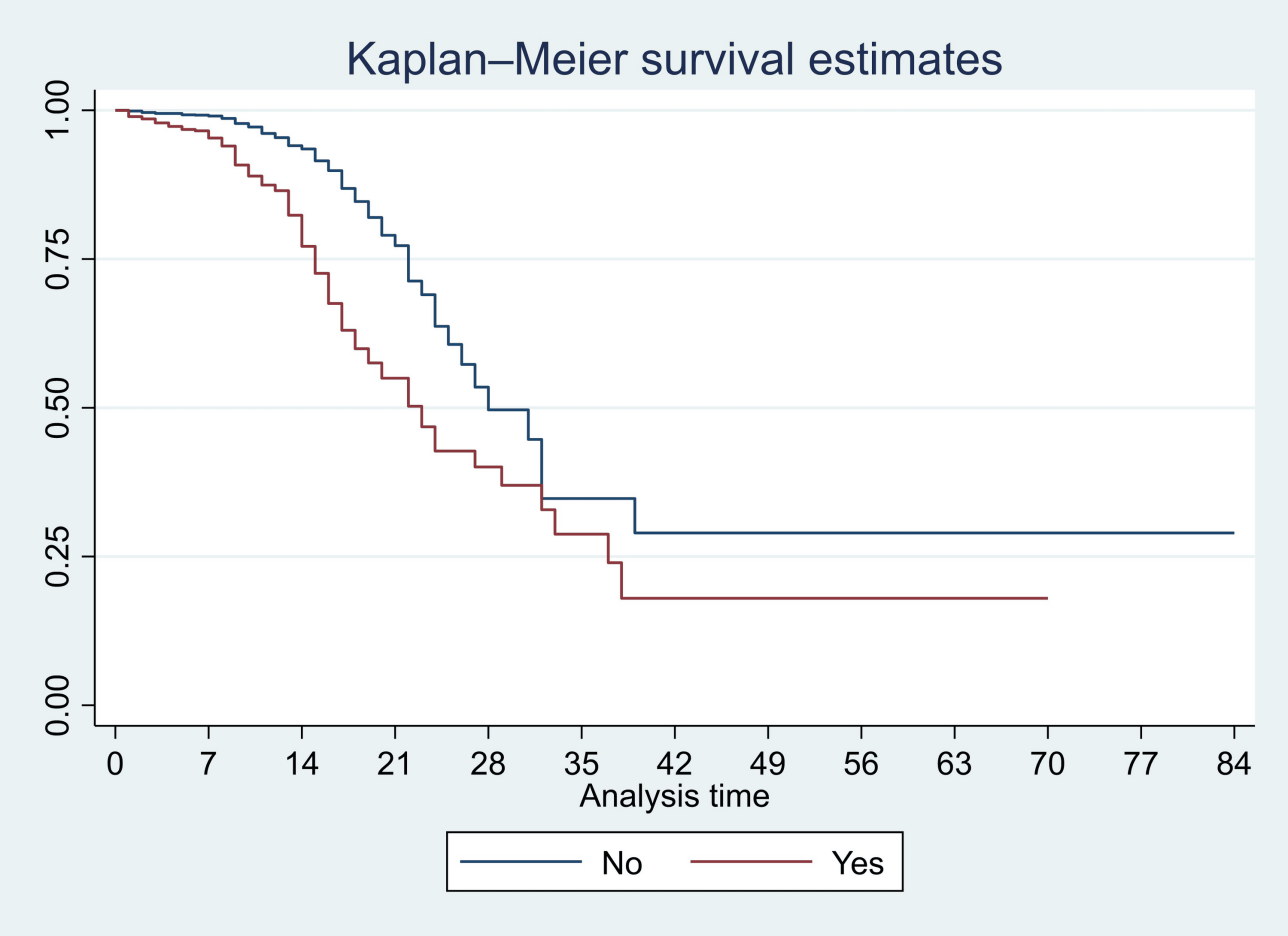
Kaplan-Meier survival curves in 3561 patients admitted with COVID-19 in a tertiary care hospital according to presentation with breathing difficulties in Mumbai, India

**Figure 5:**
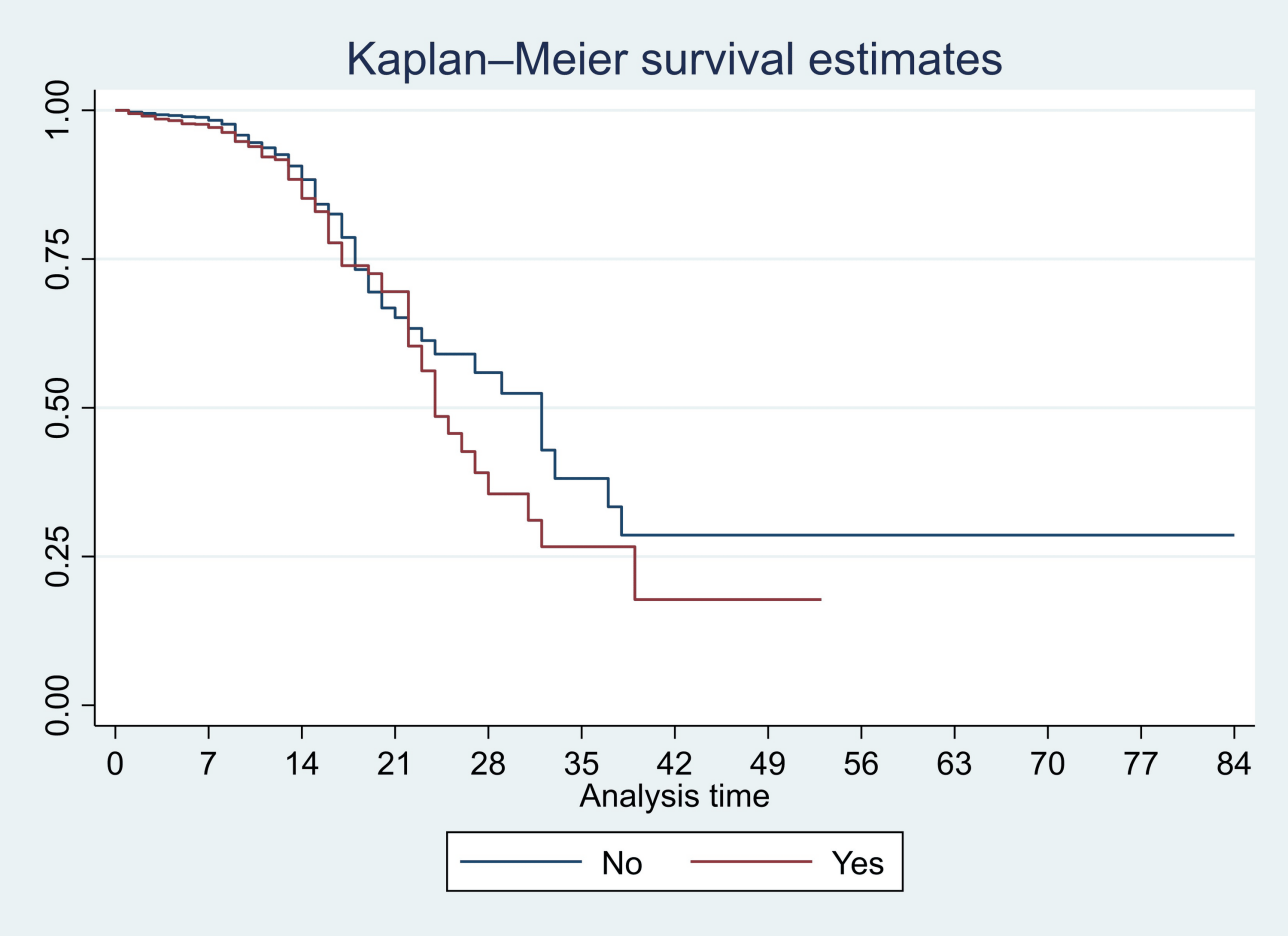
Kaplan-Meier survival curves in 3561 patients admitted with COVID-19 in a tertiary care hospital with diabetes mellitus (comorbidity) in Mumbai, India

**Figure 6:**
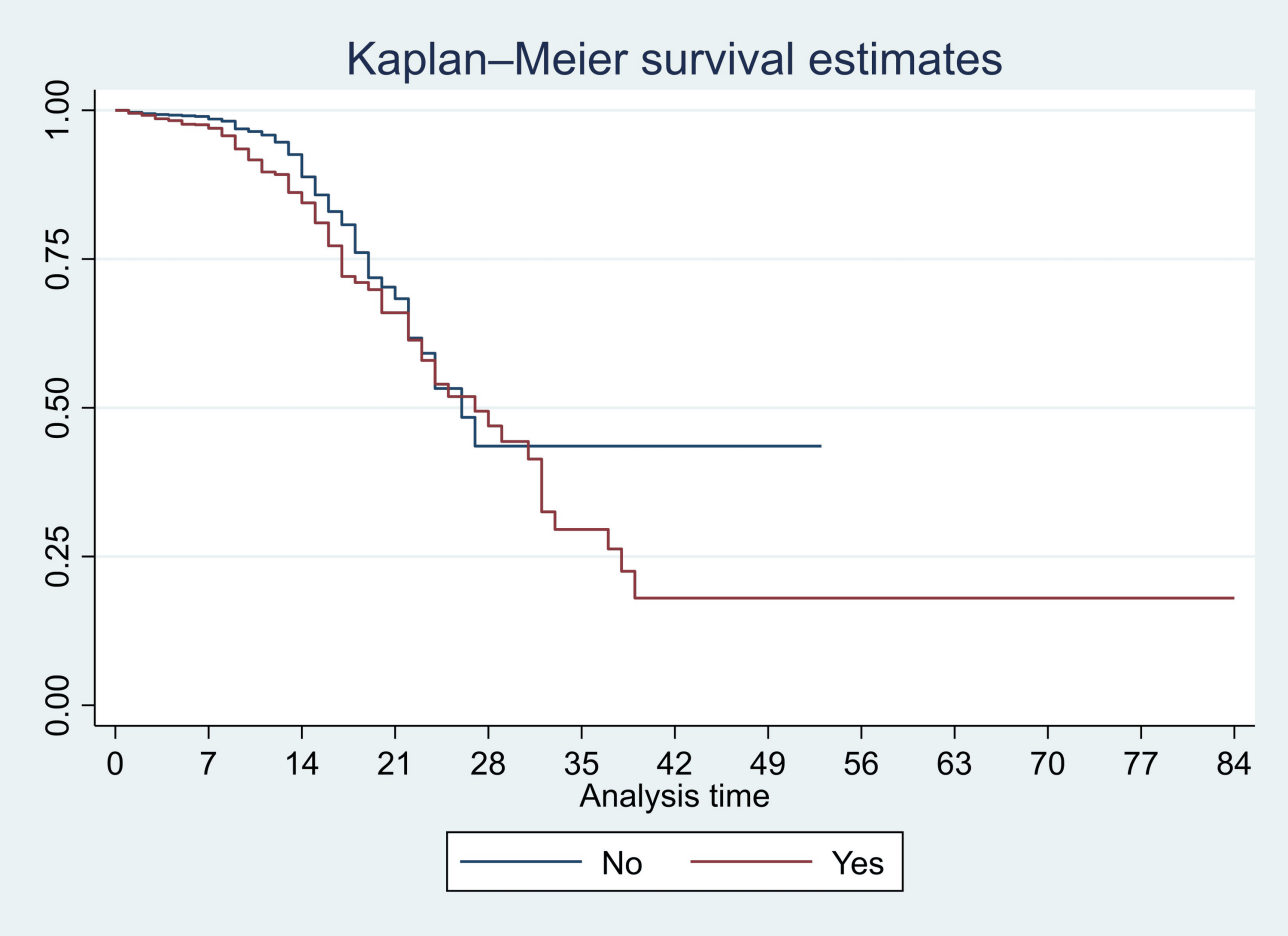
Kaplan-Meier survival curves in 3561 patients admitted with COVID-19 in a tertiary care hospital with hypertension (comorbidity) in Mumbai, India

**Figure 7:**
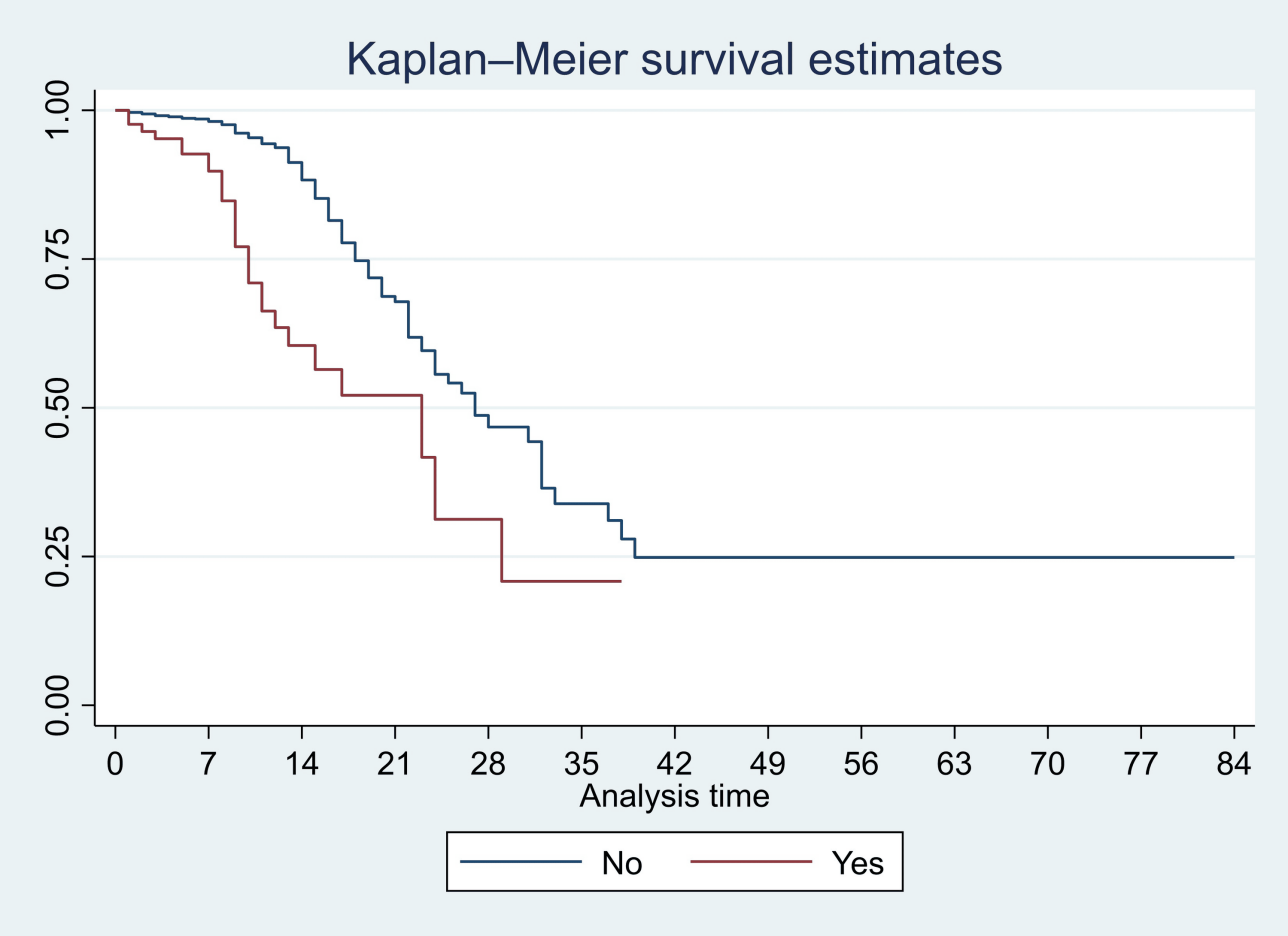
Kaplan-Meier survival curves in 3561 patients admitted with COVID-19 in a tertiary care hospital with chronic kidney disease (comorbidity) in Mumbai, India

**Figure 8:**
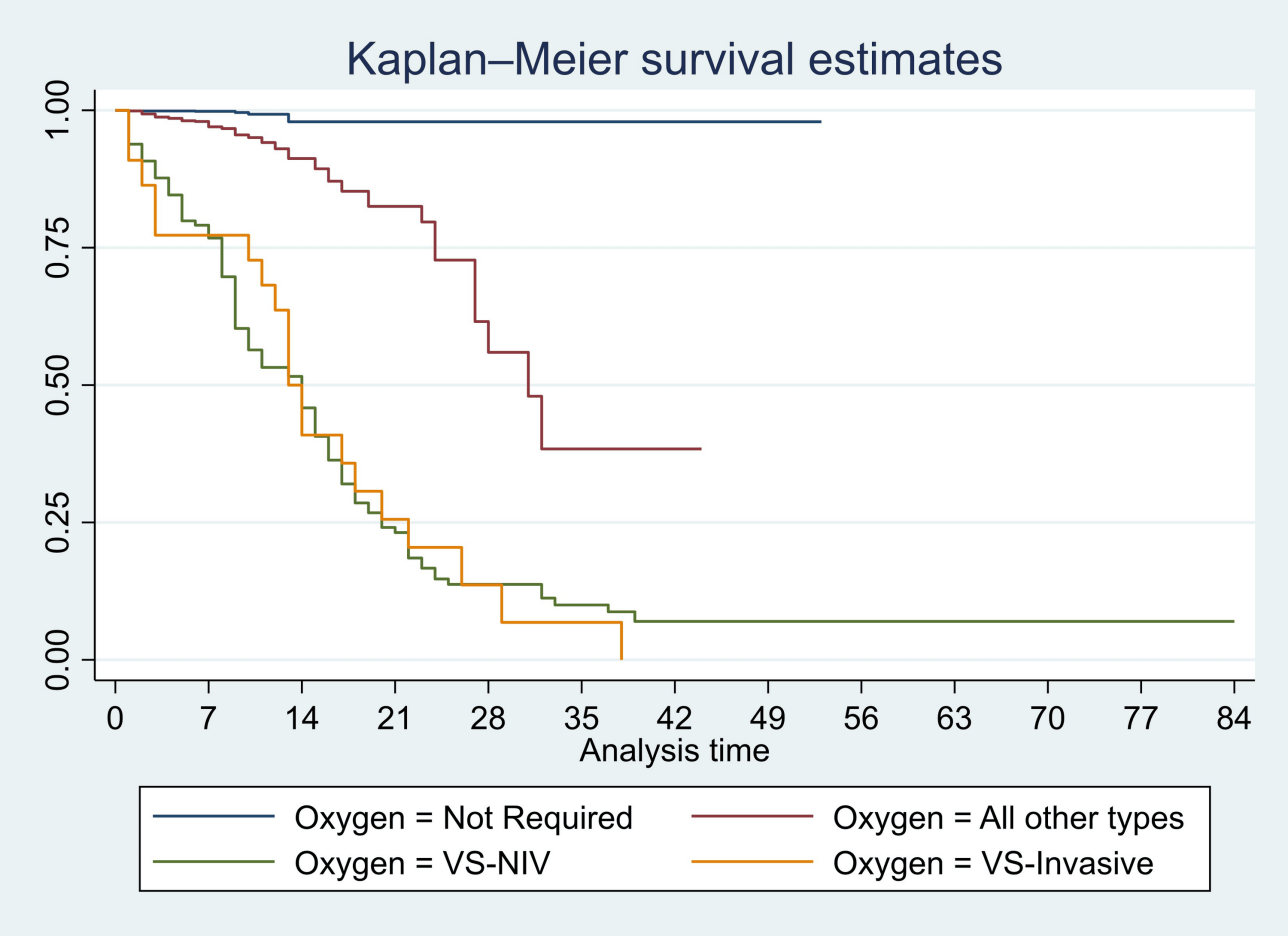
Kaplan-Meier survival curves in 3561 patients admitted with COVID-19 in a tertiary care hospital according to oxygen requirement in Mumbai, India VS-NIV: noninvasive ventilation; VS-invasive

We have presented Kaplan-Meier curves in Figures 3-8. As seen in Figure 3A, the survival was lower in individuals aged >=80 years (particularly in the first 14 days of admission) (p<0.001). There were no significant differences in the survival curves of male and female patients (p=0.26) (Figure 3B). However, survival was lower in patients who presented with breathing difficulties; the curves diverged from day one (p<0.001) (Figure 4). Similarly, survival was lower in those with diabetes mellitus, hypertension, and chronic kidney disease (Figures 5, 6, 7). In our study population, those who required ventilation support (invasive and non-invasive) had significantly lower survival compared with those who did not require ventilation or oxygen; the survival curves diverged from day one itself (p<0.001) (Figure 8).

The mortality rate (MR) was 2.03 (95% CI: 1.47, 2.82) per 100 PD in those aged >= 80 years, 0.89 (95% CI: 0.73, 1.07) per 100 PD in those aged 60-79 years, 0.45 (95% CI: 0.34, 0.59) per 100 PD in those aged 40-59 years, and it was 0.26 (95% CI: 0.14, 0.48) in 18-39-year-olds. The mortality rates were significantly higher in those >=80 years (p<0.001) and 60-79 years (p<0.001) compared with those aged 18-39 years. However, there was no significant difference in the mortality rates of those ages 40-59 years and 18-39 years (p=0.12). The mortality rate was significantly higher in males (MR: 0.79, 95% CI: 0.67, 0.92/100 PD) compared with females (MR: 0.54, 95% CI: 0.41, 0.72/100 PD) (p=0.02). Similarly, the mortality rates were high in those with chronic kidney disease (MR: 3.08, 95% CI: 2.11, 4.49/100 PD) and chronic liver disease (MR: 3.60, 95% CI: 1.35, 9.60/100 PD). Even though many variables were significant in the unadjusted hazard models, in the adjusted models, most of these variables became insignificant. In the adjusted Cox proportional hazard models, the hazard of death was significantly higher in those aged >= 80 years compared with 18-39 years (HR: 2.45, 95% CI: 1.12, 5.36; p=0.025). However, there were no significant differences in the HR of those aged 40-59 and 60-79 compared with 18-39. There was no significant difference in the hazard between males and females (HR: 1.01, 95% CI: 0.72, 1.42; p=0.94). Among co-morbidities, we found that the HR was significantly higher in those with chronic liver disease (HR: 5.12, 95% CI: 1.78, 14.71; p=0.002). The HR was significantly lower in those who were treated with injection enoxaparin (HR: 0.46, 95% CI: 0.31, 0.69; p<0.001) and borderline in those who were treated with doxycycline (HR: 0.62, 95% CI: 0.38, 1.01; p=0.057). We have presented detailed mortality rates and unadjusted and adjusted HRs in Table 4.

**Table 4:** Mortality rate and hazard ratios in 3561 patients admitted with COVID-19 in a tertiary care hospital in Mumbai, India The first column shows the mortality rate per 100 person days along with the 95% confidence intervals. The second and third columns represent the unadjusted and adjusted hazard ratios (respectively) and their 95% confidence intervals. A p-value of <0.05 was considered statistically significant. † denotes p=0.057. * p<0.05; **p<0.01; PD: person days; HR: hazard ratios; CI: confidence intervals; VS-NIV: noninvasive ventilation; VS-invasive: invasive ventilation; COPD: chronic obstructive pulmonary disease

Characteristics	Mortality rate (per 100 PD)	Hazard ratios	
		Unadjusted	Adjusted
	Estimate (95% CI)	HR (95% CI)	HR (95% CI)
Demographics			
Age			
18-39	0.26 (0.14, 0.48)	Reference	Reference
40-59	0.45 (0.34, 0.59)	1.31 (0.66, 2.59)	1.14 (0.56, 2.32)
60-79	0.89 (0.73, 1.07)	1.97 (1.02, 3.80) *	1.69 (0.84, 3.37)
>=80	2.03 (1.47, 2.82)	4.29 (2.11, 8.71) **	2.45 (1.12, 5.36) *
Gender			
Female	0.54 (0.41, 0.72)	Reference	Reference
Male	0.79 (0.67, 0.92)	1.20 (0.87, 1.65)	1.01 (0.72, 1.42)
Comorbidities (Yes)			
Asthma	0.79 (0.36, 1.77)	1.05 (0.46, 2.36)	0.88 (0.38, 2.03)
COPD	0.93 (0.35, 2.49)	1.48 (0.55, 4.00)	1.32 (0.47, 3.74)
Diabetes mellitus	0.92 (0.76, 1.13)	1.32 (1.00, 1.75) *	1.09 (0.81, 1.47)
Hypertension	1.01 (0.84, 1.20)	1.65 (1.24, 2.20) **	1.03 (0.75, 1.42)
Chronic kidney disease	3.08 (2.11, 4.49)	3.07 (2.04, 4.64) **	0.89 (0.54, 1.48)
Heart disease	1.28 (0.88, 1.84)	1.58 (1.07, 2.35) **	1.17 (0.78, 1.78)
Chronic liver disease	3.60 (1.35, 9.60)	5.69 (2.11, 15.40) **	5.12 (1.78, 14.71) **
Medications (Yes)			
Doxycycline	0.57 (0.47, 0.68)	0.55 (0.42, 0.73) **	0.62 (0.38, 1.01) ^†^
Ivermectin	0.57 (0.47, 0.68)	0.57 (0.43, 0.75) **	1.18 (0.71, 1.95)
Lopinavir/Ritonavir	2.47 (1.88, 3.24)	3.25 (2.36, 4.48) **	1.20 (0.75, 1.90)
Hydroxychloroquine	1.13 (0.88, 1.45)	1.93 (1.43, 2.63) **	1.76 (1.18, 2.63) **
Remdesivir	0.51 (0.42, 0.62)	0.40 (0.30, 0.53) **	0.81 (0.53, 1.24)
Methylprednisolone	0.91 (0.77, 1.09)	1.44 (1.08, 1.93) *	1.30 (0.92, 1.85)
Inj tocilizumab	2.40 (1.87, 3.05)	1.80 (1.30, 2.51) **	0.79 (0.54, 1.16)
Inj enoxaparin	0.52 (0.44, 0.62)	0.28 (0.21, 0.37) **	0.46 (0.31, 0.69) **
Plasma therapy	1.45 (0.78, 2.70)	1.09 (0.57, 2.08)	1.19 (0.61, 2.31)
Oxygen therapy			
Not required	0.04 (0.02, 0.09)	Reference	Reference
All other types	0.68 (0.54, 0.88)	12.94 (5.89, 28.48) **	11.16 (5.00, 24.92) **
VS-NIV	5.80 (4.82, 7.00)	91.42 (41.71, >100) **	64.56 (28.57, >100) **
VS-invasive	6.32 (4.08, 9.81)	94.26 (39.10, >100) **	74.66 (30.34, >100) **

Subgroup analysis

We did a subgroup analysis to assess the mortality according to clinical presentation and oxygen requirement. Among patients who did not present with breathing difficulties, the HR was significantly lower in patients who were treated with doxycycline (HR: 0.41, 95% CI: 0.17, 0.99; p=0.05) and injection enoxaparin (HR: 0.31, 95% CI: 0.14, 0.69, p=0.004). In those patients who presented with breathing difficulties, the HR was significantly lower in patients who were treated with remdesivir (HR: 0.52, 95% CI: 0.31, 0.90; p=0.019) and enoxaparin injection (HR: 0.58, 95% CI: 0.35, 0.95; p=0.031).

**Figure 9:**
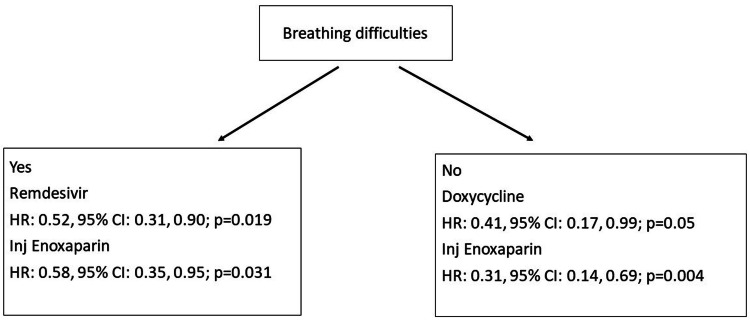
Hazard ratio for various treatment modalities according to clinical presentation (breathing difficulties) in 3561 COVID-19 patients in Mumbai, India

**Figure 10:**
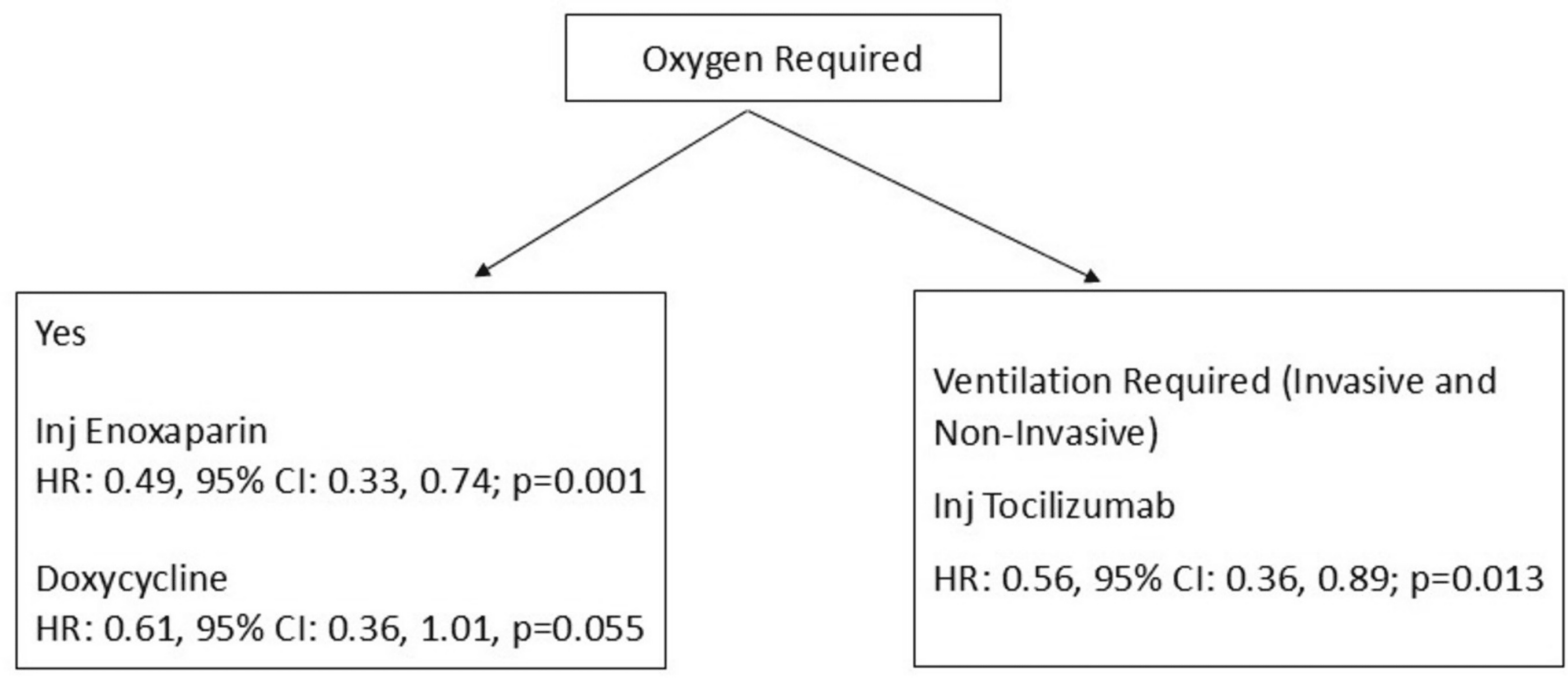
Hazard ratio for various treatment modalities according to oxygen requirement in 3561 COVID-19 patients in Mumbai, India

Among patients who required oxygen, the hazard ratio was significantly lower in patients who were treated with injection enoxaparin (HR: 0.49, 95% CI: 0.33, 0.74; p=0.001) and borderline in those who were treated with doxycycline (HR: 0.61, 95% CI: 0.36, 1.01; p=0.055). Among those patients who required ventilatory support (invasive and non-invasive, considered as severe presentation), the hazard ratio was significantly lower in patients who were treated with tocilizumab injection (HR: 0.56, 95% CI: 0.36, 0.89; p=0.013). In this same group, the hazard was significantly higher in those who were obese compared with those who were not (HR: 11.4, 95% CI: 1.1, >100, p=0.039) (Figures 9, 10).

## Discussion

Thus, in this cohort, the overall mortality in patients with COVID-19 infection admitted to a tertiary care hospital in Mumbai (India) over the initial one and a half years of the pandemic was 5.6%; the mortality rate was 0.71 per 100 PD. The deaths were higher in the initial days of the pandemic, and the proportion remained nearly stable over the next year. Mortality was higher in those who presented with breathlessness, had chronic liver disease, and required oxygen, particularly those who required invasive ventilatory support. Enoxaparin injection was an effective reduction of mortality, in general, in these patients. However, in less severe cases (patients who did not present with breathing difficulties), doxycycline was associated with a significant reduction in mortality. In patients who presented with breathing difficulties, remdesivir appeared to be useful in the reduction of mortality. Tocilizumab injection was useful in significantly reducing the mortality in patients with severe clinical presentation (patients who required ventilatory support).

In our study, mortality was about five percent over this period. This proportion is comparable to that reported by authors from other parts of India. A study by Bobdey and colleagues reported that mortality was 5.8% in a tertiary care hospital in Pune (a region close to Mumbai, India) [26]. However, other studies have reported higher mortality. For instance, a study in moderate to severe COVID-19 patients found that the 28-day mortality was 14.4% [27]. Another study by Marimuthu found the mortality to be about 10.1% [28]. Some other international studies have reported the mortality to range from 5% to 22% [29, 30]. In our study, the mortality was highest in the initial months of the pandemic; this was the time when the treatment and diagnostic protocols were just being formulated by the relevant authorities in India. As indicated earlier, we have covered nearly three waves of the pandemic in Mumbai and India during this period. The mortality was high in the initial months (wave 1) with a slight increase in a couple of months during wave 2 (delta wave), which was considered to be the more severe form, including infection in a younger population compared with wave 1 [31,32]. A higher ICU requirement and mortality were also reported by other authors from India during wave 2 [33]. Some factors associated with mortality were age, breathlessness at the time of presentation, co-morbidities (liver disease), and individuals who required oxygen (indicating a more severe form of the infection). These factors have also been associated with higher mortality in other studies [34-37]. In addition, obesity has been considered a high-risk factor for mortality by various authors [38]. Helvaci and colleagues have suggested that obese patients may be at a higher risk for severe forms of infection, which may be associated with the progression of the disease [39]. Zhang and co-workers, however, reported that though obesity may be associated with a more severe form of COVID-19, it is not associated with mortality [40]. Finally, Huang and co-authors reported that obesity is associated with severity and mortality in COVID-19 patients [41]. In our analyses, obesity was associated with mortality only in those who had the severe form of the disease.

Management of COVID-19 patients was another important aspect of these analyses. We found that enoxaparin injection was associated with reduced mortality in the whole cohort as well as in various subgroups (those who presented with breathlessness/did not present with breathlessness, those who required oxygen). It has been suggested that enoxaparin is useful in COVID-19 not only because of its anticoagulant properties but also because it reduces the inflammatory markers, neutrophil extracellular traps, and organ dysfunction [42]. Clinical studies have shown mixed results, with some trials suggesting that thromboprophylaxis with enoxaparin did not reduce deaths in patients with COVID-19, whereas other studies have shown that enoxaparin was associated with lower thrombotic events and mortality [43-45]. A systematic review reported that therapeutic and prophylactic anticoagulant regimens reduced in-hospital mortality, a finding that was also seen in our study [46]. These may be a useful option for patients who present with thromboembolic events; these may be associated with mortality in these patients [47]. Use of doxycycline was associated with lower mortality in patients who did not present with breathlessness (probably indicating a less severe form of COVID-19). Doxycycline was considered as a treatment option due to its anti-viral, anti-inflammatory, and immunomodulatory effects [48,49]. An open-labeled community trial of doxycycline in older individuals and those with comorbidities found no significant reduction in hospitalization or mortality [50]. However, they did find that the proportion of intensive admissions and duration of the hospital stay was lower in the group in which doxycycline was used, even though the difference was not statistically significant [50]. Hence, these authors concluded that doxycycline should not be used for regular management of COVID-19.

Some of the other drugs that have been useful in the management of COVID-19 are remdesivir and injection tocilizumab. Remdesivir was one of the first antivirals approved for the treatment of COVID-19 by the United States Federal and Drug Administration [51]. Beigel and colleagues in a large, randomized trial found that remdesivir was useful in shortening the time to recovery in adults with COVID-19 and lower respiratory tract infection [52]. Another meta-analysis found that remdesivir was useful in the treatment of COVID-19 patients who were non-ventilated and required supplemental oxygen [53]. We found that remdesivir was useful in reducing mortality in patients who presented with breathing difficulties. Finally, tocilizumab was the other medication that reduced the mortality in severely ill patients who required ventilatory support. Previous literature on tocilizumab has been mixed. Some authors found that tocilizumab did not have any major effect on clinical outcomes such as time to recovery or mortality in patients who were treated with tocilizumab [54]. However, a study by Siami and colleagues found that tocilizumab reduced the mortality in these patients by preventing end-organ damage and reduction of advanced life support [55]. Finally, a meta-analysis of 17 randomized trials found that administration of tocilizumab was associated with a significant reduction in mortality in these patients [56].

We have presented our experience of the management of COVID-19 patients who presented in the first one and a half years of the pandemic in Mumbai, India. The patients were treated by multiple physicians based on the existing protocol, guidelines, clinical knowledge, and presentation of the patients. This was not a part of any trial; thus, patients may have received multiple medications at the same time. Some medications may have been stopped, and others started based on clinical features. Though we used multivariate analysis, the sequence of medication may not have been accounted for in these models. This is a potential limitation of the study. We have not presented the laboratory findings of these patients. Since these were done on the prescription of the treating physician, based on the clinical indication, some of these laboratory parameters were not consistent across all patients. The study, however, provides useful information on clinical presentation and factors associated with mortality in COVID-19 infections. The study also presents the experience in the management of these patients over the time period of the pandemic in Mumbai, India.

## Conclusions

In general, we found that the overall mortality over these 20 months was 5.6%. The mortality was highest during the initial days of the pandemic and in patients with comorbidities. After the initial days of the pandemic, once the protocols and guidelines were introduced and followed, the mortality rate stabilized relatively and increased slightly during the second wave. The oxygen requirement was high during the peak of the COVID-19 waves and waned when the number of total admissions reduced. Remdesivir and tocilizumab injection were useful in the reduction of mortality in the severe form of COVID-19 infection. Doxycycline was useful in milder and less severe forms of infection. However, enoxaparin injection was associated with lower mortality in most of these cases. Thus, this may be a useful treatment option for patients presenting with COVID-19 infection, since thromboembolic events may be common and associated with mortality in these patients.
